# The effect of source claims on statement believability and speaker accountability

**DOI:** 10.3758/s13421-021-01186-x

**Published:** 2021-06-14

**Authors:** Johannes B. Mahr, Gergely Csibra

**Affiliations:** 1 Department of Psychology, Harvard University, 33 Kirkland St, Cambridge, MA 02138 USA; 2 Department of Cognitive Science, Central European University, Budapest, Hungary; 3 Department of Psychological Sciences, Birkbeck, University of London, London, UK

**Keywords:** Source claims, Evidentials, Speaker commitment, Statement believability

## Abstract

What is the effect of source claims (such as “I saw it” or “Somebody told me”) on the believability of statements, and what mechanisms are responsible for this effect? In this study, we tested the idea that source claims impact statement believability by modulating the extent to which a speaker is perceived to be committed to (and thereby accountable for) the truth of her assertion. Across three experiments, we presented participants with statements associated with different source claims, asked them to judge how much they believed the statements, and how much the speaker was responsible if the statement turned out to be false. We found that (1) statement believability predicted speaker accountability independently of a statement’s perceived prior likelihood or associated source claim; (2) being associated with a claim to first-hand (“I saw that . . .”) or second-hand (“Somebody told me that . . .”) evidence strengthened this association; (3) bare assertions about specific circumstances were commonly interpreted as claims to first-hand evidence; and (4) (everything else being equal) claims to first-hand evidence increased while claims to second-hand evidence decreased both statement believability and speaker accountability. These results support the idea that the believability of a statement is closely related to how committed to its truth the speaker is perceived to be and that source claims modulate the extent of this perceived commitment.

Unlike most other animals, human beings form a large part of their beliefs without relying on direct personal experience. Instead, we acquire many of our beliefs socially, through the testimony of others. The capacity for social information transmission allows us to learn about places we have never visited and about times we have never lived through. It also allows us to form beliefs that are too abstract (“The distance from the earth to the moon is 384,499 km”) or opaque (“God is the unity of a Trinity”) to ever be formed on the basis of personal experience alone (Gergely & Csibra, 2011). However, even though human communicative capacities might look like a super power from the perspective of other species lacking such abilities, they have various cognitive ‘conditions of possibility’ (Sperber et al., 2010). In particular, in order for such a complex system of cooperative communicative exchange of information to be evolutionarily stable, both speakers and listeners need to benefit from this exchange. On the one hand, speakers require mechanisms to influence a listener’s mind in the absence of trust. On the other hand, listeners need to be able to determine when to rely on messages from speakers who might not always have an interest in transmitting true information.

## Speaker commitment can stabilize human communication

One device which has the potential to solve this dilemma is commitment (e.g., Brandom, 1983; Geurts, 2019). If speakers can signal different degrees to which they are willing to take responsibility for the truth of their messages, and listeners can track these signals, this might support the stabilization of communication. By signaling the extent to which they are ready to bear either direct or reputational consequences in case they are found to be wrong, speakers might allow listeners to gauge how much to rely on the truth of the claim in question (Bonalumi et al., 2019). In essence, the strength of a speaker’s commitment might be viewed as a signal for how much she is willing to ‘bet’ on the truth of her assertion. On the one hand, this allows listeners to accept claims they might otherwise reject on the guarantee of the speaker. On the other hand, it allows speakers to ‘hedge their bets’ so as to avoid responsibility for a potentially false claim.

From this perspective, the believability of a statement should to some extent depend on how accountable a speaker has made herself for its truth. In other words, how much people believe a given statement should be predicted by how accountable they hold a speaker when they find out that the statement was wrong. Such an effect has been found in the investigation of various pragmatic phenomena. For example, expressions of confidence or certainty (Lorson et al., 2021; Vullioud et al., 2016), different pragmatic devices for encoding speaker meaning (Mazzarella et al., 2018), and modal expressions (Degen et al., 2019) have all been shown to modulate perceived speaker commitment.

## The role of source claims in communication

However, one device that has so far not been explicitly investigated from this perspective is source claims (i.e., claims about the origin of the information in question). This is surprising given that source claims seem to have a large impact on how believable a statement is perceived to be (Mercier, 2020). When deciding what to believe, listeners do not only pay attention to the content of the message and the identity of the speaker (Collins et al., 2018; Sperber et al., 2010), but also to what evidence a speaker purports to have (Koenig, 2012). Even preschoolers have been shown to preferably believe assertions supported by claims to first-hand evidence over claims supported by circular arguments (Castelain et al., 2016; Mercier et al., 2014). The effect of source information on believability should be particularly familiar to members of institutions dealing in ‘knowledge production’, such as academia and journalism. Here, accurate source reporting is conceived of as a central, ethical requirement for good practice. A claim that at first sight might seem uncontroversial can be undermined when attributed to an untrustworthy source. Conversely, a claim that might seem unlikely can become believable in association with an authoritative source (e.g., Sperber et al., 2010). In particular, ‘having been there’ seems to convey such authority beyond mere expertise. In fact, qualitative research methods (such as ethnography) might be said to be based to a large extent on the authority that first-hand experience conveys.

While the role of sources in institutionalized forms of knowledge production is particularly prominent, source information plays an important role in everyday communication, too. For example, source claims have been identified to play an important role in the transmission of rumors and gossip (Altay & Mercier, 2020; Caplow, 1947; DiFonzo & Bordia, 2002). Moreover, source claims can be used to claim credit, hedge one’s bets, or increase believability (Altay, Claidière, & Mercier, 2020; Altay, Majima, & Mercier, 2020; Shaw & Olson, 2015).

The pronounced effects of source information in communication have arguably led to its grammaticalization in at least one quarter of all recorded languages as ‘evidentials’ (e.g., Aikhenvald, 2004, 2014, 2018). However, even languages that do not encode source claims through explicit grammatical markers have a multitude of ways to express evidentiality. For example, modal expressions in English and German (such as *must*, *presumably*, *maybe, definitely*) have an evidential interpretation (Degen et al., 2019). In fact, the role of source information in communication is so important that Mahr and Csibra (2018; 2020) have proposed that it contributed to shaping the evolution of episodic memory to serve as a form of source memory in humans. Mahr and Csibra explicitly argued that source memory plays such an important role in communication because it allows speakers to effectively modulate their conversational commitments and thereby greatly expand their communicative competencies.

## Intuitions about the differential reliability of sources

More generally, humans reason about different sources of information and have strong intuitions about their differential reliability (Papafragou et al., 2007; Ünal & Papafragou, 2018). In particular, we seem to regard direct perceptual evidence as more reliable than inferential and reportative evidence. *Ceteris paribus*, claims to more direct sources of evidence (such as perception) intuitively increase the believability of a statement, while claims to more indirect sources of evidence (such as hearsay) decrease its believability. Of course, whenever a specific second-hand source is specified, this relationship might not hold (your doctor might know more about the cause of your symptoms than you do in spite of your direct experience of them). And clearly, the identity of the speaker herself will make a large difference to the effect of her evidential claim (if you’re known to be a pathological liar, I won’t believe you, even if you claim to have first-hand knowledge). Nonetheless, second-hand information is often regarded as less reliable than first-hand information qua being second-hand. This intuition is also reflected in legal history: many legal systems (such as in medieval Europe, ancient Athens, or ancient Hindu law) have historically forbidden hearsay as a source of evidence (Mercier & Boyer, 2021). Similarly, in epistemology a fundamental disagreement divides ‘reductionists’ such as Hume (see also, e.g., Adler, 2002; Van Cleve, 2006) and ‘antireductionists’ such as Reid (see also, e.g., Coady, 1992; Hardwig, 1985) over the question whether testimony by itself provides sufficient justification for belief or whether we need additional positive reasons to be justified in accepting information transmitted via testimony.

Such intuitions about the differential reliability of different sources have been shown to emerge in childhood. For example, 3-year-olds already seem to take second-hand evidence to be less reliable than direct evidence (Robinson & Whitcombe, 2003). Further, Fitneva (2008) showed that Bulgarian 6-year-old to 9-year-old children judged that a third party would be more likely to believe statements marked by perceptual and first-hand evidentials compared with statements marked by inferential and reportative evidentials (for evidence from Turkish, see Ozturk & Papafragou, 2016). Similarly, Lane et al. (2018) recently found that 6-year-olds to 8-year-olds preferred claims to first-hand evidence in belief formation about improbable events. However, this effect only emerged when claims to first-hand evidence were contrasted with claims to second-hand evidence. Claims to first-hand evidence alone did not change children’s beliefs.

In contrast, in adults the effect of explicit evidential claims on statement believability has not received much attention through experimental research. Wilson, Wilczynski, Wells, and Weiser (2000) found that eyewitness accounts increased the credibility of statements compared with second-hand accounts only in scenarios that were of high social importance. But these authors did not measure how different evidential claims impacted perceived speaker commitment. Collins and Hahn (2019) recently found that indirect evidential claims such as “I suspect that . . .” did not allow speakers to ‘hedge their bets’; that is, speakers asserting a falsehood paired with an indirect source claim still received a hit to their reputation in the eyes of their listeners. However, this study did not investigate direct or reportative evidential claims and did not include assessments of the impact of evidential claims on statement believability.

## Source claims and their relationship to speaker commitment

While the intuition that statements based on first-hand evidence are generally more believable than statements based on second-hand evidence is widespread (Faller, 2001), it is less clear why it should hold. In particular, the current study was designed to distinguish between two possible mechanisms.

First, it is possible that the effect of evidential claims on statement believability is due to purely ‘epistemic’ factors. That is, listeners might assume that different kinds of sources are ordered according to a ‘scale of reliability’ (Faller, 2001, 2002). Such assumptions might be rooted in a tacit theory about how information behaves when it ‘travels’. Listeners might believe that, since perception is most directly causally connected to the world, beliefs formed on its basis should be most likely to be true. In contrast, if a piece of information is not acquired by perception directly but instead through inference from perception, chances for informational degradation increase. As a consequence, listeners might assume that, *ceteris paribus*, any inferential ‘step’ a given piece of information is removed from direct perception has the potential to degrade the quality of said information. In the case of claims based on second-hand evidence, information has to be perceived, communicated, received, and communicated again in order to arrive at the listener. If listeners indeed have a tacit theory about informational degradation through inference, and if listeners implicitly track how many inferential steps a piece of information traversed to arrive at them, they should arguably rely less on assertions based on second-hand evidence compared with first-hand evidence (everything else being equal). In essence, on this view, evidential claims influence statement believability in a similar way as arguments do: by providing a reason (in the form of a source claim), which might allow the listener to weigh the strength of the evidence herself.

Even though it is possible that people do indeed reason about source information in this way (i.e., as arguments) this cannot entirely explain why claims to first-hand evidence should *ceteris paribus* be held to be more believable than claims to second-hand evidence. First, note that source claims do not function like other reasons or arguments in the sense that they do not merely leave it up to an audience to come to the same conclusion as the speaker on the basis of impartial evidence. At least in the case of claims to first-hand evidence, a source claim must to some extent come down to the audience’s trust in the speaker. As such, if claiming first-hand evidence exclusively impacted an audience’s beliefs through appealing to people’s intuitions about the differential reliability of sources, it would leave such an audience open to manipulation. What would keep a manipulative speaker from regularly purporting to have first-hand evidence in order to increase her believability? In other words, if claims to first-hand evidence indeed increase a statement’s believability, they arguably can only do so at a potential cost to the speaker.

Thus, a second way of explaining the differential effect of claims to direct versus indirect forms of evidence might be through *social factors*. On standard accounts of the speech act of asserting (Brandom, 1983; Geurts, 2019), making an assertion commits the speaker to the truth of a proposition. Listeners monitor what a speaker commits herself to and take these commitments as signals for the reliability of whatever the speaker is asserting. Source claims might therefore have differential effects on how reliable their assertion is perceived to be not solely through listeners’ assumptions about how perception and report generally affect the reliability of testimony. Instead, source claims might impact statement believability also by modulating what additional commitments a speaker is viewed to undertake.^1^ That is, while making an assertion always produces a commitment to a given proposition, embedding a proposition in an evidential clause might modulate this commitment or produce additional commitments. For example, when a speaker makes a claim to direct evidence (e.g., “I saw that *p*”), listeners might take her to be committed to both *p* (‘seeing’ being a factive expression) as well as to *knowing that p*. In contrast, when a speaker makes a claim based on reportative evidence (e.g., “Somebody told me that *p*”), she might not be taken to be committed to *p* but only to the fact that she was *told that p*. As a consequence, while claims to direct evidence will commonly commit the speaker to the truth of the embedded proposition, claims to indirect evidence might not (or at least not directly).

Evidential claims might therefore be another means through which speakers can signal what they are committed to. In fact, source claims might be particularly effective in this regard. After all, when a speaker claims to have first-hand information, she implicitly accepts *direct* responsibility for the truth of her assertion. If a speaker claiming to have first-hand evidence is found to be wrong, she cannot defer responsibility to anything but the failing of her perceptual system or memory. In contrast, if a speaker claims to have second-hand evidence for a proposition, she will always be able to (partially) defer responsibility for its truth to this second-hand source. As such, she should be perceived to be differently committed than in the case of having claimed first-hand evidence.^2^ Thus, on this view, source claims do not exclusively impact statement believability through their function as arguments but can also function as commitment signals (akin to modal claims such as expressions of confidence, e.g., Vullioud et al., 2016).

Indeed, speakers seem to be intently sensitive to these differential social effects of their evidential claims. Speakers have been shown to flexibly adjust their source claims in service of their communicative goals. For example, Altay, Cladiére, and Mercier (2020) recently showed that speakers in a transmission chain experiment were biased towards reporting their source for rumors as being a ‘friend of a friend’, thereby optimizing believability while minimizing accountability. Giardini and Conte (2012) similarly reported ethnographic evidence suggesting that speakers adaptively avoid being seen as the source of gossip in order to avoid accountability. In contrast, Castelain et al. (2019) have shown that members of Ecuadorian small-scale societies were likely to present information as if it had been acquired by them personally rather than revealing the second-hand source of their belief when it was beneficial to do so.

Similar arguments have been discussed in the literature on evidentials in the context of the question to what extent evidential markers only convey information about source or whether they also convey other information, such as speaker commitment (e.g., Aikhenvald, 2004, 2018; Chafe, 1986). In particular, it has been debated whether evidential and modal expressions (i.e., expressions of certainty) should be categorically distinguished. For example, Faller (2002) proposed that one way to draw the distinction is by pointing to the fact that, while evidentials semantically encode information source, epistemic modals merely *imply* it. Conversely, epistemic modals might be said to encode different speaker commitments while evidentials merely imply them. Regardless of one’s stance on this question, however, it has been observed that, just as modal expressions, evidential claims can have effects on speaker commitment (e.g., Wierner, 2018; see Degen et al., 2019, for experimental evidence in this regard). Framed in these terms then, the question we are asking here is whether differential effects on reliability judgments of different evidential claims can be accounted for purely through semantically encoded information (i.e., assumptions about the properties of the respective source) or whether inferences to speaker commitment play a role as well.

## The present study

Crucially, having undertaken a commitment should be particularly apparent when the commitment is viewed to have been broken. Therefore, one way to measure what a speaker is viewed to be conversationally committed to is to test how listeners react when certain aspects of her assertion turn out to be false. By asking participants how much they hold a given speaker accountable for ‘being wrong’ we can assess to what extent they took that speaker to be committed to the proposition in question. As such, the view taking evidential claims to function exclusively as arguments does not predict a straight-forward connection between their effect on statement believability and speaker accountability. If source claims affect the believability of a statement purely through appealing to listeners’ intuitions about the effects of different sources on a statement’s reliability, how accountable the speaker is viewed to be for the truth of her assertion should not be modulated by source claims.

In contrast, viewing source claims as commitment devices predicts that such claims should modulate both the extent to which a statement is believed *and* how accountable a speaker is viewed for its truth. The main goal of the present study was therefore to differentiate between these two accounts by testing whether claims to first-hand and second-hand evidence differentially impact the relationship between statement believability and speaker accountability. After all, if it is indeed the case that claims to first-hand but not second-hand evidence commit the speaker to having knowledge about the proposition in question, this difference should affect how accountable listeners hold a speaker in each case if the proposition turns out to be false.

More specifically, we predicted that, if source claims serve as commitment devices, being associated with a source claim should increase how strongly a statement’s believability predicts the speaker’s accountability. This should be true even though claims to first-hand and second-hand evidence should impact believability and accountability in opposite directions. If a speaker purports to have direct evidence for the truth of a given proposition, this should increase both how believable her statement is viewed to be *and* how accountable the speaker is taken to be in case the embedded proposition is found to be false. In contrast, claims to reportative evidence should have the opposite effect.

Thus, in the current study, we set out to (1) test whether claims to first-hand and second-hand evidence differ in their effects on statement believability, and (2) whether analogous effects can be found on how accountable a speaker is viewed for the truth of their assertion.

**Fig. 1:**
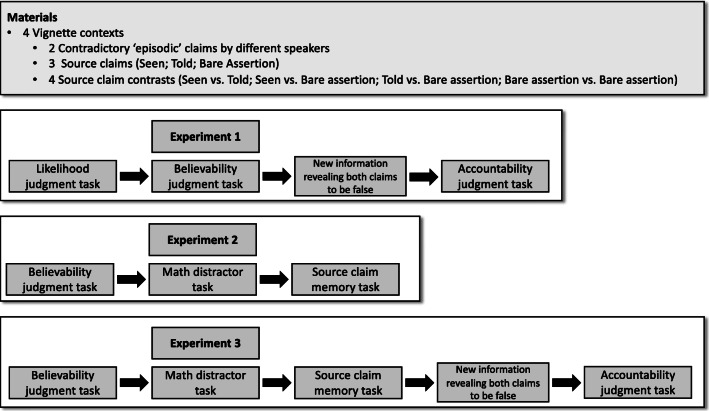
Task structure for Experiments 1 through 3

We investigated these questions in three experiments (see Fig. 1). In Experiment 1, we sought to determine (1) whether claims to first-hand evidence and claims to second-hand evidence impact statement believability and speaker accountability in opposite directions, (2) whether a relationship between statement believability and speaker accountability exists, (3) whether this relationship is impacted by the modulation of speaker commitment that making a source claim should entail, and (4) how these effects interact with a statement’s prior likelihood. To do so, we carried out an online, vignette-based experiment in which participants were presented with two contradictory statements by different speakers about the same specific past event in four different vignette contexts. Participants completed three tasks on these materials.

First, participants were presented with a ‘Likelihood judgment task’: each statement was presented without an associated source claim or speaker, and participants were asked to evaluate how likely they perceived it to be.

Next, participants were asked to complete a ‘Believability judgment task’: they were presented with each vignette context and the associated statement again, however, this time each statement was presented as being asserted by a different speaker, each making either a source claim about how they came to know about what they were stating or merely asserting the statement. Speakers claimed to have information about the event based either on (1) having seen it (“I saw that . . .”), (2) having been told (“Somebody told me that . . .”) or (3) without an explicit source claim. Participants were then asked to rate how much they believed each of the speakers.

Finally, participants were presented with each vignette context and the associated statements (with the respective evidential claims) a third time and asked to complete an ‘Accountability judgment task’: for each vignette context, they received new information which revealed that, in fact, both statements had been false. For example, in one of the vignette contexts both speakers claimed that the Boston Celtics had lost their last game to the Los Angeles Lakers (each claiming a different point differential). In the second phase of the experiment, it was revealed that, in fact, the Celtics had beaten the Lakers. Participants were then asked to make ‘accountability judgments’, that is, to report how much they blamed each speaker for having made a false statement (“How much do you blame [speaker] for misleading you?”).

One issue of relevance in this context is the question how evidential claims affect different kinds of assertions (Stephens & Koenig, 2015). On the one hand, claims about a well-specified spatiotemporal context (‘episodic claims’) might be well suited to be justified and supported through claims to direct experience. The fact that I saw that Peter kissed Mary, might allow me to speak authoritatively about the fact that, indeed, Peter kissed Mary. On the other hand, it is less clear what the effect of claims to direct experience should be on claims about generics (‘semantic claims’). After all, the fact that I saw Peter kissing Mary might, for example, not allow me to speak with the same authority about whether they are a couple or not. Making the claim that “I saw that Peter and Mary are a couple” might instead be interpreted as having evidence through inference. To avoid these complications, we restricted our experimental materials exclusively to episodic claims: assertions about particular, past events.

Our main predictions here were as follows. First, regarding source claim effects on believability, we predicted that claims to first-hand evidence (‘seen’ claims) would increase, while claims to second-hand evidence (‘told’ claims) would decrease statement believability compared with bare assertions. Second, regarding source claim effects on the relationship between believability and accountability, we predicted that, when a given statement was associated with a ‘seen’ or ‘told’ claim, believability and accountability judgments should be more highly correlated than when a statement was barely asserted. A more detailed description of these predictions can be found below.

Next, in Experiments 2 and 3 we investigated the relationship between bare assertions and claims to first-hand evidence. In particular, we investigated whether participants pragmatically interpret bare assertions as ‘seen’ claims (for a similar effect see, e.g., Degen et al., 2019) with the use of a memory confusion paradigm (see Fig. 1). Note that, on the account outlined above, both bare assertions and claims to direct evidence commit the speaker to the truth of the embedded proposition. Moreover, a bare assertion for an episodic claim might be taken to imply that the speaker has good evidence for the truth of that proposition. Hence, bare assertions and claims to first-hand evidence might be difficult to distinguish based on believability and accountability ratings alone. For this reason, we asked whether participants would sometimes encode bare assertions as claims to first-hand evidence and would therefore be more likely to recall bare assertions as ‘seen’ claims than vice versa. This allowed us to then test the same predictions as in Experiment 1: Experiment 2 asked whether, controlling for similarities in interpretation, ‘seen’ claims were associated with higher believability ratings than bare assertions, and Experiment 3 allowed us to ask whether an analogous effect could be found in accountability judgments.

## Experiment 1

**Fig. 2:**
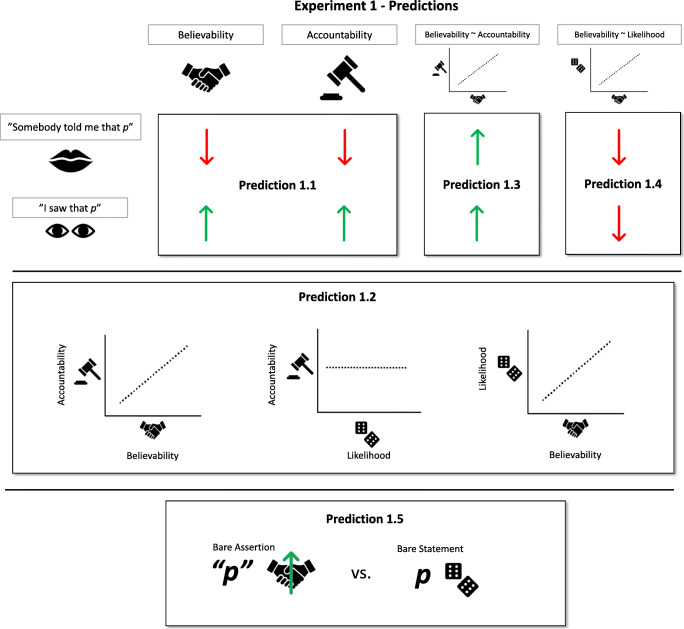
Graphical depiction of the predictions for Experiment 1. Predictions for the effect of source claims (i.e., Prediction 1.1, 1.3, and 1.4) are depicted in relation to the “bare assertion” baseline condition

Experiment 1 made the following predictions (see Fig. 2).

- *Prediction 1.1:* Claims to first-hand evidence (‘having seen’) should increase while claims to second-hand evidence (‘having been told’) should decrease the believability of a statement relative to bare assertions.
- *Prediction 1.2:* Accountability judgments should be predicted by how believable participants took a statement to be, but not by how likely they judged it to be true.
- *Prediction 1.3:* This association between believability and accountability should be impacted by our source claim factor: A statement being associated with a claim to first-hand or second-hand evidence should increase the extent to which believability and accountability ratings are associated compared with statements being merely asserted.
- *Prediction 1.4:* The association between believability and likelihood ratings should depend on source claims: A statement being associated with a claim to first-hand or second-hand evidence should decrease the extent to which believability and likelihood ratings are associated compared with statements being merely asserted.
- *Prediction 1.5*: Merely asserting a statement should increase its believability compared with how likely it was judged to be prior to being associated with such an assertion.

To motivate Prediction 1.2, consider that making an assertion is likely to commit a speaker to the truth of a proposition and therefore should make them somewhat accountable in case it is found to be false. Thus, believability and accountability should be correlated across all source claim conditions. In contrast, there should be no such relationship between accountability and how likely participants perceive a given statement to be. This should be the case in spite of the fact that perceived likelihood should have a strong influence on how believable a given statement is taken to be. That is, both accountability and likelihood should be related to believability without being related to each other.

However, the association between accountability and believability should be increased whenever a speaker actively modulates their commitment. Prediction 1.3 captures this idea: If source claims indeed modulate believability in virtue of their effect on speaker commitment, we should find increased correlation between believability and accountability for statements associated with an explicit source claim compared with statements not associated with such a claim. Crucially, again, this relationship should not be explained by how likely a statement is taken to be. While likelihood judgments should capture how much participants believe a certain statement based on their prior knowledge, believability judgments should capture how much participants believe that statement when asserted by a speaker. As such, the variability of accountability ratings should explain the variability of believability ratings (Prediction 1.2) but not necessarily that of likelihood ratings (Prediction 1.4).

As a corollary of this prediction, we might expect that merely asserting a statement might increase a statement’s believability compared with how likely it was judged without having been asserted by anyone: making an assertion should increase believability not just because asserting is committal but also because asserting a claim provides evidence to a listener that at least one other person believes the claim in question (Prediction 1.5.)

Note that, due to the correlational nature of the current design, and in spite of these predictions, we will not be able to make causal claims about the relationship between statement believability and speaker accountability. The causal arrow could thus go in both directions: On the one hand, participants might compute speaker commitment when they are exposed to a given statement and calibrate their believability judgments on this basis.^3^ On the other hand, believability might drive perceived speaker accountability. In this case, participants might perceive speakers to be more accountable for statements that they ‘were made to believe’. While the predictions of the current study were derived from the former account, we will not be able to rule out the latter. In either case, however, source claims would have—either directly or indirectly (via their effect on believability)—an impact on speaker accountability.

## Methods

### Participants

We recruited 509 native-English speaking participants online using Amazon Mechanical Turk. If participants provided the same response to all questions for at least one of our measures (believability or accountability judgments), we took this as an indication that they did not complete the task correctly and excluded them from our analysis. Eighty-two participants were excluded this way, leaving 427 participants in the final sample (mean age = 33.51 years, *SD* = 6.23, 203 females). All experiments reported here were approved by Central European University’s United Ethical Review Committee for Research in Psychology (EPKEB).

### Design

The structure of the task and the materials for Experiment 1 are summarized in Fig. 1. Vignette contexts and the associated statements were constructed to ensure that both statements referred to the same spatiotemporal context (e.g., the same game of basketball) while being mutually exclusive (i.e., both statements could not be true at the same time).

After participants had completed a consent form, they were instructed that they would read a number of vignettes and make likelihood judgments about each statement presented by itself (“How likely do you think it is that [Statement 1]”; “How likely do you think it is that [Statement 2]”; 1 = *not at all likely*; 6 = *very likely*). Participants were then presented with the same vignettes and statements again, however, the two statements were now associated with different speakers and source claims, and believability judgments were requested about each. After having completed all believability judgments (“How much do you believe [Speaker 1]?”; “How much do you believe [Speaker 2]?” 1 = *not at all*; 6 = *completely*), participants were then presented with each vignette again in the same order (including each statement) and received new information about the context, which revealed that the statements of both speakers had in fact been wrong. For each vignette, participants were then asked to judge how much they hold each speaker accountable for being wrong (“How much do you blame [Speaker 1] for misleading you?”; “How much do you blame [Speaker 2] for misleading you?” 1 = *not at all*; 6 = *completely*). We did not tell participants about the subsequent judgments so as to avoid any interference effects between these different judgments.

Thus, Experiment 1 had two nested factors: (1) what source claim a given individual statement was associated with (‘seen’, ‘told’, ‘bare assertion’) and (2) which two source claims were contrasted with one another in a given trial (‘seen vs. told’; ‘seen vs. bare assertion’; ‘told vs. bare assertion’; ‘bare assertion vs. bare assertion’; see Fig. 1). Given that in any given trial two contradictory statements were contrasted with one another, our analysis focused on the ‘source contrast’ factor. Our dependent measures were Likelihood (as assessed by the likelihood question), Believability (as assessed by the believability question) and Accountability (as assessed by the accountability question). All judgments were recorded on a 6-point Likert scale (1 = *not at all*; 6 = *completely*).

Even though each participant was exposed to each vignette and each statement, we counterbalanced which source claim was associated with which statement, the order in which vignette contexts were presented, as well as which source contrast was associated with which vignette across participants. Thus, across participants each vignette was associated with each source contrast and each individual statement was associated with each source claim.

### Materials

Each vignette consisted of a short description of an everyday occurrence accompanied with a picture depicting the described situation (the full stimulus set of vignettes can be found here: https://osf.io/yzpsh/?view_only=886a9467643946f784ced6f23a494b07). The following is an example of one of the vignettes with the accompanying statements in each task.

Vignette context:

You are a big fan of the Boston Celtics basketball team. Unfortunately, you missed their last game against the Los Angeles Lakers because you were on the road to visit your family. When you arrive, you ask your brothers **David** and **Carl** how the game went.

[Picture depicting the context]

Likelihood judgment task:

Please evaluate how likely it would be that:

The Lakers beat the Celtics 105 to 92. [1 = not at all likely; 6 = very likely]

The Lakers beat the Celtics by two points. [1 = not at all likely; 6 = very likely]

Believability judgment task:

David answers: “[I saw that / somebody told me that / no source claim] the Lakers beat the Celtics 105 to 92.”

Carl answers: “[I saw that / somebody told me that / no source claim] the Lakers won by 2 points.”

[How much do you believe David? 1 = not at all; 6 = completely]

[How much do you believe Carl? 1 = not at all; 6 = completely]

Accountability judgment task:

For this vignette, participants subsequently received the following new information on which to base their accountability judgments:

When you check the results of the game on your phone before going to bed, you find out that, contrary to what your brothers told you, the Boston Celtics actually beat the Lakers 108 to 103.

[How much do you blame David for misleading you?]

[How much do you blame Carl for misleading you?]

## Results

Because our experimental design exposed the participants to contrasts between two statements associated with different source claims, our analysis primarily focused on the source contrast factor. Thus, we computed difference scores for each source contrast while treating ‘bare assertions’ as a baseline. That is, we computed the difference between believability/accountability ratings in a given trial in the following way:

1. ‘Seen’ statement—‘Told’ statement
2. ‘Seen’ statement—Bare Assertion
3. ‘Told’ Statement—Bare Assertion
4. Bare Assertion—Bare Assertion

**Fig. 3:**
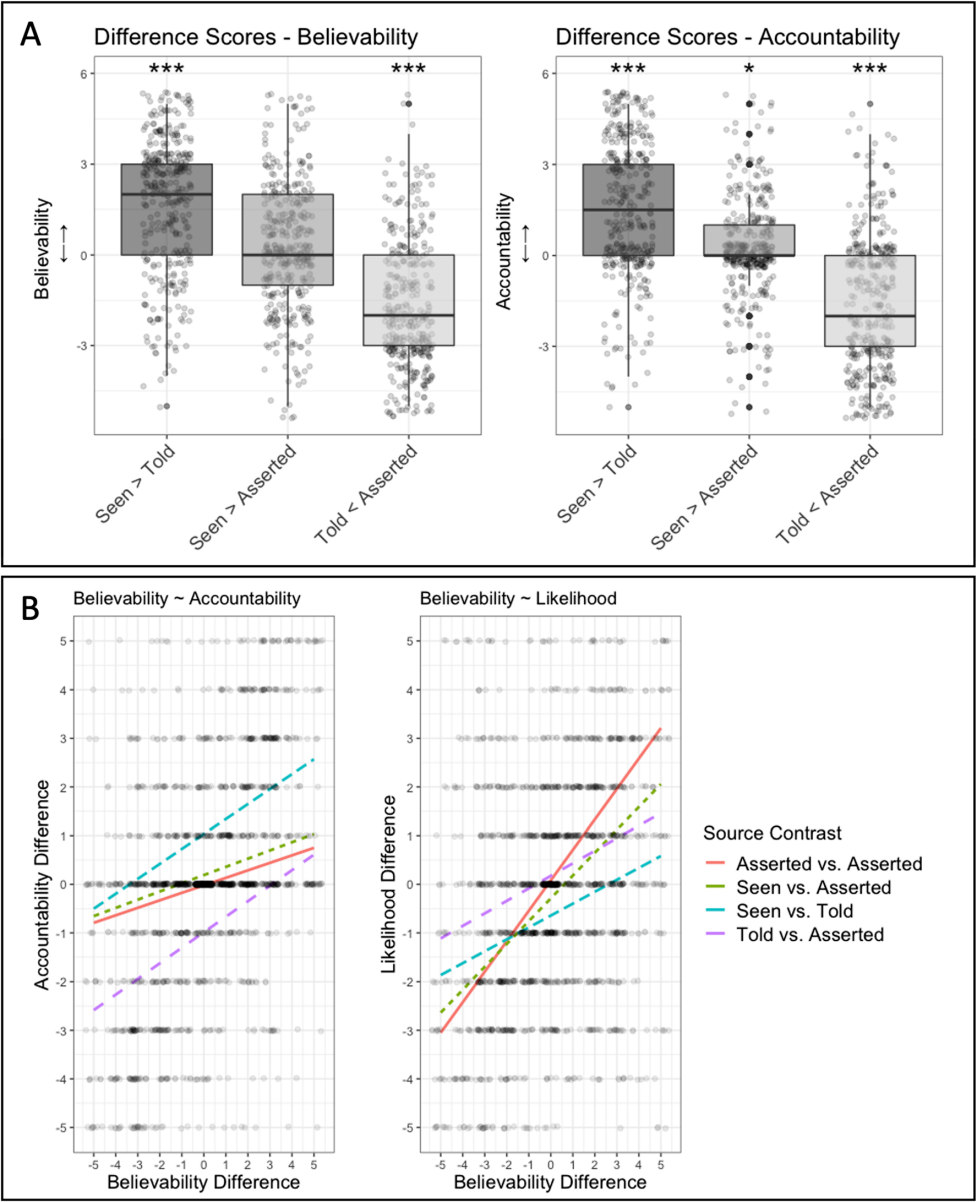
The results of Experiment 1. **a** Difference scores in each source contrast conditions. Significance stars indicate the results of one-sample t tests against 0. While scores differed from 0 in the Seen vs. Told and Told vs. Asserted conditions for both believability and accountability ratings, scores in the Seen vs. Asserted conditions did not differ from 0 in the believability task and differed only weakly in the accountability task. **b** Left: Results of linear regression for the relationship between believability and accountability difference scores depending on source contrast. Scatter plots depict individual difference scores. Compared with baseline (Asserted vs. Asserted), difference scores were more strongly correlated in the Seen vs. Told and Told vs. Asserted, but not the Seen vs. Asserted conditions. Right: Results of linear regression for the relationship between believability and likelihood difference scores depending on source contrast. Compared with baseline, difference scores were less strongly correlated in the Seen vs. Told and Told vs. Asserted, but not the Seen vs Asserted conditions

In addition to believability and accountability judgments, we also calculated difference scores for likelihood ratings following the same logic (i.e., by subtracting rating values according to which source claim condition a given statement would be associated with in the subsequent believability and accountability judgment tasks). Because we counterbalanced which statement was associated with which source claim in two counterbalancing orders across participants, the above logic of computing difference scores meant that, for half of our participants ratings for the first statement in a given trial were subtracted from the ratings for second statement, while for the other half of participants the opposite was the case. Thus, difference scores for (4) were calculated by subtracting ratings for the first statement from those for the second statement in counterbalancing Order 1 and vice versa in counterbalancing Order 2. Each participant contributed one such difference score per source contrast condition (see Fig. 3a).^4^ All analyses for this and the subsequent experiments were carried out in R (R Core Team, 2020) and RStudio (RStudio Team, 2020), plots were generated with *ggplot2* (Wickham, 2016), and mixed-effects regression models were computed with *lme4* (Bates et al., 2015).

### Prediction 1.1: What was the effect of source claims on believability and accountability ratings?

In order to test whether ratings differed within source contrasts, we computed one-sample *t* tests (with a Bonferroni-corrected alpha-level of .0083 (.05/6); see Fig. 3a) comparing difference scores in each source contrast to 0 for both believability and accountability ratings. While believability as well as accountability difference scores in both ‘seen vs. told’ trials (*M*_Believability_ = 1.75, *SD* = 2.22, t_Believability_ = 15.2, *p*_Believability_ < .0001; *M*_Accountabiilty_ = 1.57, *SD* = 2.24, *t*_Accountabiilty_ = 13.5, *p*_Accountabiilty_ < .0001) and ‘told vs. bare assertion’ trials (M_Believability_ = −1.73, SD = 2.06, t_Believability_ = −16.1, *p*_Believability_ < .0001; *M*_Accountabiilty_ = −1.54, *SD* = 2.23, *t*_Accountabiilty_ = −13.3, *p*_Accountabiilty_ < .0001) differed from 0, in ‘seen vs. bare assertion’ trials only accountability difference scores (*M*_Accountabiilty_ = 0.23, *SD* = 1.64, *t*_Accountabiilty_ = 2.74, *p*_Accountabiilty_ = .0064) and not believability difference scores (*M*_Believability_ = 0.25, *SD* = 2.1, , *t*_Believability_ = 2.32, *p*_Believability_ = .021) differed from 0.

### Predictions 1.2 and 1.3: Was the relationship between believability and accountability impacted by source claims independently of likelihood?

To determine the extent to which believability and accountability judgments were associated with one another independently of perceived likelihood (Prediction 1.2) and to assess whether our source claim manipulation had an effect on this relationship (Prediction 1.3), we computed a linear regression model.^5^ We used accountability difference scores as the dependent variable and entered likelihood difference scores as well as the interaction between believability difference scores and source contrast condition as predictors. We used a treatment coding scheme for the source contrast factor specifying the ‘bare assertion vs. bare assertion’ condition as the reference level (see Fig. 2b). This model, *F*(8, 1486) = 80.85, Δ*R*^2^ = 0.3, *p* < .0001, showed (in line with Prediction 1.2) that believability ratings were associated with accountability ratings in the baseline condition (β = 0.13, *SE* = 0.05, *p* = .012, 95% CI [0.03, 0.24]). As predicted, accountability judgments were not affected by likelihood judgments (β = 0.03, *SE* = 0.02, *p* = .155, 95% CI [−0.01, 0.78]).

Further, partially confirming Prediction 1.3, the relationship between accountability and believability differences was modulated by our source claim manipulation such that when a statement was associated with a ‘told’ claim, believability responses predicted accountability responses (β_Belief*Seen-Told_ = 0.17, *SE* = 0.07, *p* = .014, 95% CI [0.03, 0.3]; β_Belief*Told-Asserted_ = 0.18, *SE* = 0.07, *p* = .011, 95% CI [0.04, 0.31]) more strongly than in the ‘bare assertion vs. bare assertion’ baseline contrast. However, contrary to what we predicted, ‘seen’ claims did not have an effect on the association between believability and accountability difference scores (β_Belief*Seen-Asserted_ = 0.02, *SE* = 0.07, *p* = .774, 95% CI [−0.12, 0.15]) beyond that found in the baseline condition.

### Prediction 1.4: Was the relationship between believability and likelihood impacted by source claims?

Next, we tested whether source contrast modulated the extent to which believability difference scores were predicted by likelihood difference scores (see Fig. 3b). As stated in Prediction 1.4, if our source claim manipulation affected the extent to which believability judgments depended on factors other than mere perceived likelihood, we would expect there to be an interaction effect between likelihood ratings and source contrast: The presence of a source claim should reduce the extent to which believability ratings were predicted by likelihood ratings alone compared with the ‘bare assertion vs. bare assertion’ reference level. To do so, we computed another linear regression model, with believability difference scores as the outcome variable, and predictors for accountability difference scores as well as the interaction between source contrast and likelihood difference scores. Again, the source contrast factor was entered into the model via a treatment coding scheme specifying the ‘bare assertion vs. bare assertion’ condition as the reference group.

In line with the above prediction, the model, *F*(8, 1486) = 122.3, Δ*R*^2^ = 0.39, *p* < .0001, showed that even though likelihood differences predicted believability differences in the baseline condition (β_Likelihood_ = 0.41, *SE* = 0.04, *p* < .0001, 95% CI [0.32, 0.49]), when one of the statements was associated with a ‘told’ claim, likelihood difference scores were less predictive of believability difference scores than in the baseline condition (β_Likelihood*Seen-Told_ = −0.15, *SE* = 0.06, *p* = .022, 95% CI [−0.27, −0.02]; β_Likelihood*Told-Asserted_ = −0.23, *SE* = 0.06, *p* < .0001, 95% CI [−0.35, −0.12]). However, the relationship between likelihood and believability difference scores was not modulated in the ‘seen vs. asserted’ condition compared with the ‘bare assertion vs. bare assertion’ baseline (β_Likelihood*Seen-Asserted_ = −0.05, SE = 0.06, *p* = .36, 95% CI [−0.17, 0.06]).

### Prediction 1.5: Did being asserted increase a statements’ believability beyond its perceived likelihood?

Finally, to assess whether merely being asserted increased a statement’s believability compared with its prior likelihood, we computed a paired-sample *t* test comparing likelihood and believability ratings for statements that were associated with a bare assertion. This test suggested that believability ratings for asserted statements (*M*_Belief-Asserted_ = 3.73, *SD* = 1.07) were higher than likelihood ratings for the same statements presented without an associated speaker (*M*_Likelihood-Asserted_ = 3.6, *SD* = 1.01), *t*(426) = 6.99, p < .0001.

## Discussion

Experiment 1 had two main goals. First, we wanted to determine whether claims to first-hand evidence increase, while claims to second-hand evidence decrease both statement believability and speaker accountability. Second, we sought to test whether the effect of evidential claims on statement believability can be explained through their effect on speaker accountability.

Regarding the first goal (Prediction 1.1), evidence from Experiment 1 was mixed. While claims to second-hand evidence reliably reduced both statement believability and speaker accountability, the effect of claims to first-hand evidence was less clear. In particular, bare assertions behaved similarly to claims of first-hand evidence. While it is possible that claims to first-hand evidence do not increase believability and accountability compared with bare assertions, another possibility is that participants interpreted bare assertions as claims to first-hand evidence. In other words, because the protagonists in our materials made claims about specific past events that they could have (in fact, should have) experienced themselves, participants might have assumed that they must have based their assertion on first-hand evidence. Participants might therefore have treated speakers who only implied to have first-hand evidence in the same way as speakers who explicitly claimed to have such evidence. This finding would be in line with work showing that listeners often take speakers to be committed to their implied meaning (Bonalumi et al., 2020).

Regarding the second goal, Experiment 1 confirmed that differences in accountability judgments predicted differences in believability judgments independently of differences in likelihood judgments (as stated in Prediction 1.2). Moreover, we found evidence that merely asserting a statement increased the statement’s believability compared with its prior likelihood (as stated in Prediction 1.5)^6^. Both of these findings are to be expected if making an assertion minimally commits a speaker to the truth of her assertion and are not specifically related to the effect of source claims.

Results regarding the effect of source claims on the relationship between believability, accountability, and likelihood judgments were mixed. Recall that we predicted that being associated with a source claim would increase the association between believability and accountability (Predictions 1.3) while decreasing the association between likelihood and believability (Predictions 1.4). However, our results suggest that only ‘told’ claims followed this pattern: while difference scores in source contrasts in which one of the statements was associated with a ‘told’ claim showed a higher association between believability and accountability judgments and a lower association between believability and likelihood judgments (compared with baseline), we did not find the same effect for ‘seen’ claims.

What could explain this outcome? On the one hand, of course, it is possible that ‘seen’ claims do indeed not influence how strongly a statement’s believability is tied to how accountable the speaker is taken to be for its truth. However, on the other hand, if participants indeed interpreted bare assertions as claims to first-hand evidence it is to be expected that we would not find an increase in the association between believability and accountability for ‘seen’ claims over bare assertions. Thus, Experiments 2 and 3 were designed to test whether participants in fact interpreted ‘seen’ statements and bare assertions as claims to the same forms of evidence and whether this could explain the lack of difference between claims to first-hand evidence and bare assertions in Experiment 1.

## Experiment 2

In Experiment 1, claims to first-hand evidence and bare assertions were hard to distinguish in terms of their impact on statement believability and speaker accountability. Why did claims to first-hand evidence not differ more strongly in their effect on believability and accountability?

One possibility is that assertions about episodic claims might have been implicitly interpreted as claims to first-hand evidence by our participants. People might assume that whenever a speaker does not make an effort to explicitly specify her source about an episode that plausibly occurred to her directly, she must have first-hand evidence. On this view, first-hand evidence might be seen as the ‘default’ source of evidence for episodic claims such that only deviations from this default warrant explicit mention. If this was the case, the fact that we did not find stronger differences between ‘seen’ statements and bare assertions in Experiments 1 could be explained by the fact that, in fact, participants took both kinds of statements as being associated with the same kind of source claim. Any difference we observed would then have merely been due to the explicitness of the source claim in the ‘seen’ condition.

This interpretation is also suggested by a recent study by Degen et al. (2019; see also Altay, Majima, & Mercier, 2020, Experiment 3) who found that participants interpreted bare assertions as being grounded in first-hand evidence. To test for this possibility in the current context, we developed a memory confusion paradigm with the materials from Experiment 1. After participants had made believability judgments as in Experiment 1, we presented them with each statement again in a surprise memory test for the source claim associated with each statement (‘seen’, ‘told’, ‘no source’). Since our hypotheses in this experiment did not relate to the prior likelihood of statements, we did not include a likelihood judgment task in Experiment 2.

Our predictions were as follows:

- *Prediction 2.1:* Source confusions should primarily affect bare assertions; that is, participants should be unlikely to confuse ‘seen’ and ‘told’ statements or ‘told’ statements and bare assertions. As a result, participants should be more accurate in their memory responses in the ‘seen’ than in the ‘bare assertion’ condition.
- *Prediction 2.2:* If participants encode bare assertions as ‘seen’ statements, they should be more likely to confuse bare assertions with ‘seen’ statements than vice versa.
- *Prediction 2.3:* If these memory confusions are indeed an effect of how participants pragmatically interpret statements in the believability task (rather than merely an effect of how participants recall different source claims) higher believability ratings should be associated with statements that are recalled to have been associated with a ‘seen’ claim compared with those recalled as having been barely asserted. Such an effect would also suggest that, controlling for pragmatic interpretation, claims to first-hand evidence increase a statement’s believability.

## Methods

### Participants

We recruited 196 native English-speaking participants from the online testing platform Testable Minds (https://minds.testable.org/; Rezlescu et al., 2020). As in the previous experiment, we excluded all participants who provided the same response to all questions in one of our two tasks (believability judgments or memory judgments). Thirteen participants were excluded this way so that 183 participants were included in the final sample (mean age = 33.42 years, *SD* = 8.56 years; 100 females).

### Design

Experiment 2 was identical to Experiment 1 in materials, but we removed both the likelihood and the accountability judgment tasks. Instead, after having made believability judgments, participants were asked to complete a distractor task (solving 50 simple math problems of the form “11 + 23 = ?”) before being presented with each vignette and the associated statements (without source claims) in randomized order for a surprise memory test. In this memory test, participants were asked to recall for each statement what source claim it had originally been associated with in a three-alternatives forced-choice test (‘seen’/‘told’/‘no source’; the complete task can be found here: https://www.testable.org/experiment/2878/448737/start).

## Results

**Fig. 4:**
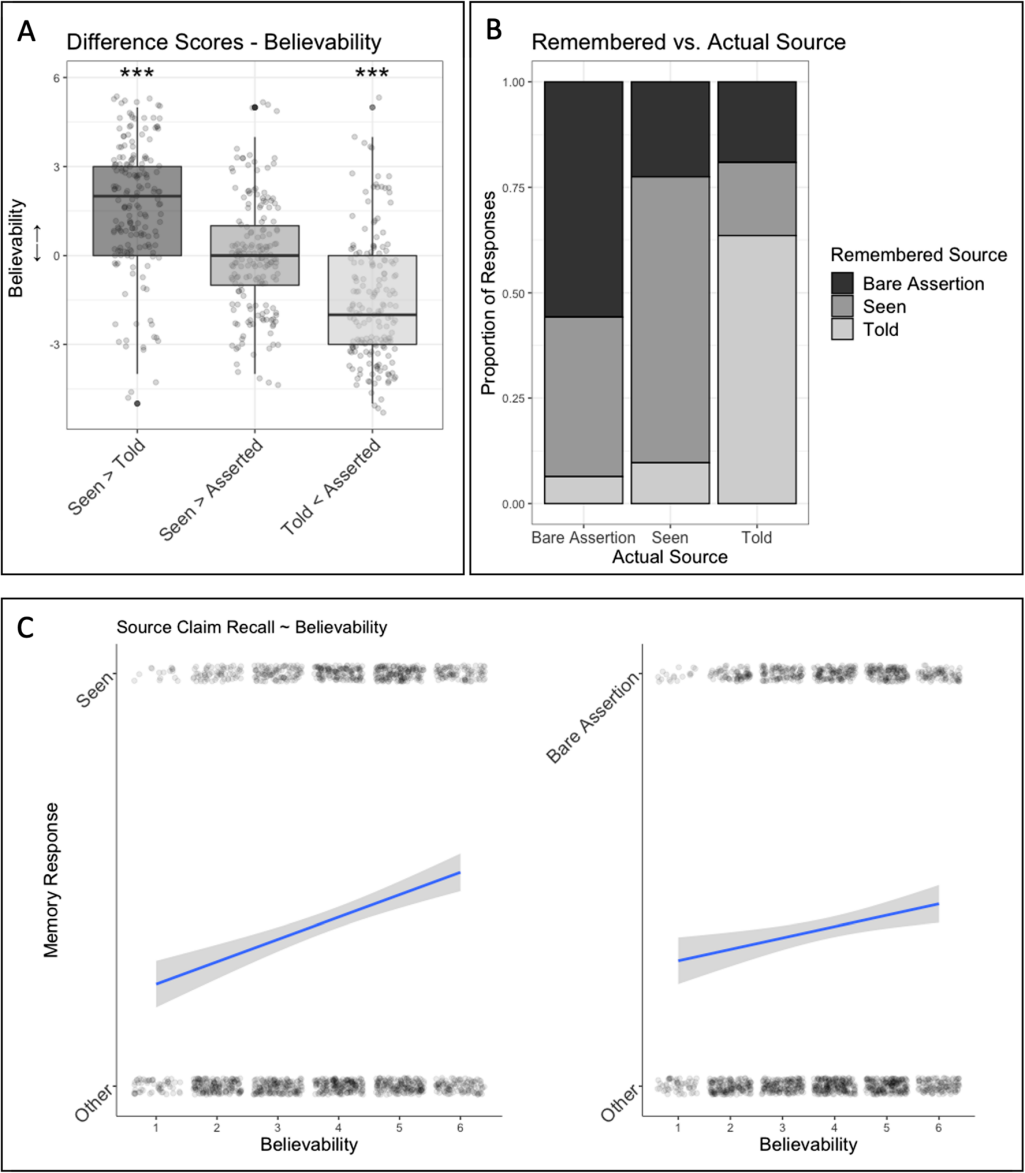
The results of Experiment 2. **a** Believability difference scores in each source contrast. **b** The distributions of remembered source claims as a function of the source claim a given statement had originally been associated with in the believability task. Participants were more likely to confuse in recall bare assertions with ‘seen’ claims than vice versa. **c** Correlations between believability ratings and ‘seen’ memory responses (left) as well as believability ratings and ‘bare assertion/no source’ memory responses (right). Believability ratings predicted 'seen' memory responses but not 'bare assertion' memory responses.

Distributions of believability and memory responses are displayed in Fig. 4a and b, respectively. Experiment 2 largely replicated the results of Experiment 1 in terms of believability ratings.

### Prediction 2.1: Were participants more accurate in recalling ‘seen’ claims than ‘bare assertions’?

To analyze participants’ source claim recall, we fit a logistic mixed-effects model to a binary variable coding for whether participants had responded correctly in the memory task (yes/no). This model included random effects for participant number and vignette number (with fixed slopes) and a fixed effect for source claim condition (entered into the model via dummy coding with ‘bare assertion’ serving as the reference level). The *p* values were obtained by likelihood ratio tests (LRTs) of the full model with the source claim effect against the model without this effect. For the estimates of the model parameters, the 95% confidence intervals were assessed by computing a likelihood profile and finding the appropriate cutoffs based on the LRT.

Model comparison suggested that, while participants performed better at recalling ‘seen’ claims (*M*_Seen-Recall_ = 0.68, *SD*_Seen-Recall_ = 0.35) than bare assertions (*M*_Asserted-Recall_ = 0.56, *SD*_Asserted-Recall_ = 0.36), χ^2^(1) = 16.82, *p* < .0001, 95% CI [0.31, 0.88], we did not find significant differences in recall performance between ‘told’ claims and bare assertions (*M*_Told-Recall_ = 0.64, *SD*_Told-Recall_ = 0.37), χ^2^(1) = 3.05, *p* = .081.

### Prediction 2.2: How likely were participants to confuse bare assertions and ‘seen’ claims in recall?

Next, to determine whether participants produced source confusions to suggest that they had encoded bare assertions as ‘seen’ statements, we compared proportions of different memory error types to chance level (0.5) via one-sample *t* tests (see Fig. 4b; with a Bonferroni-corrected alpha-level of .017 and excluding those participants who did not make memory errors in recalling a given type of source claim). This showed that memory errors in the ‘told’ condition were roughly equally distributed between ‘seen’ (*M* = 0.47, *SD* = 0.48) and ‘no source’ responses, *t*(97) = 0.53, *p* = .599. In contrast, participants were more likely than chance to recall bare assertions as ‘seen’ (*M* = 0.86, *SD* = 0.27), *t*(133) = 15.7, *p* < .0001, and less likely than chance to recall them as ‘told’. Further, participants were also more likely to confuse ‘seen’ claims with bare assertions (*M* = 0.7, *SD* = 0.45) than ‘told’ claims in memory, *t*(91) = 4.33, *p* < .0001. To test whether participants were more likely to confuse bare assertions with ‘seen’ claims than vice versa (Prediction 2.2), we calculated the proportion of confusions between bare assertions and seen claims (for each participant that committed at least one of these confusions) that was due to assertions being recalled as ‘seen’ (*M* = 0.73, *SD* = 0.36) and compared it to chance, *t*(150) = 7.69, *p* < .0001 (see Fig 4b). This suggested that participants were more likely to recall bare assertions as ‘seen’ claims than vice versa. Note also that the number of participants who did not commit any memory errors differed across source claim conditions: While only 49 (27%) participants recalled all bare assertions correctly, 85 (46%) participants recalled all told and 91 (50%) participants recalled all seen statements correctly.

### Prediction 2.3: Were believability ratings predicted by ‘seen’ or ‘bare assertion/no source’ memory responses?

Next, we tested whether participants’ believability judgments predicted whether they would remember a given statement to have been associated with a claim to first-hand evidence compared with another source claim (see Fig. 4c). To do so, we first fit a binomial logistic mixed-effects model to a binary variable coding for whether participants had responded with ‘seen’ in the memory test. We included believability judgments and statements’ ‘original source claim’ (seen/told/bare assertion) as fixed effects and participant number and vignette number as random effects with fixed slopes. LRT-based model comparison showed that, controlling for the effects of a statement’s original source, seen memory responses were predicted by participants believability ratings, χ^2^(1) = 9.94, *p* = .002, 95% CI [0.06, 0.27]. We then fit the same model to a binary variable coding for whether participants responded with ‘no source’ in the memory test. However, LRT-based model comparison suggested that believability ratings did not predict ‘no source’ memory responses, χ^2^(1) = 0.07, *p* = .934, 95% CI [−0.11, 0.10].

### Prediction 1.5: Did bare assertions still increase believability compared with prior likelihood when we control for repeated exposure?

Recall that, in Experiment 1, we found that bare assertions increased a statements believability compared with its perceived prior likelihood. However, this effect might have been caused by the fact that participants had been exposed to the same statement repeatedly. In Experiment 2, in contrast to Experiment 1, participants were exposed to each statement for the first time in the believability judgment task. Thus, in order to rule out that the believability increase associated with bare assertions over a statement’s prior likelihood found in Experiment 1 was merely due to repeated exposure, we conducted an exploratory analysis comparing believability ratings for bare assertions in Experiment 2 with likelihood ratings for those same statements in Experiment 1 via a two-sample *t* test. This test suggested that believability ratings for bare assertions in Experiment 2 (*M*_Belief-Asserted_ = 4.09, *SD*_Belief-Asserted_ = 1.34) were higher than likelihood ratings for the same statements in Experiment 1 (*M*_Likelihood-Asserted_ = 3.67, *SD*_Likelihood-Asserted_ = 1.44), *t*(316.33) = 5.70, *p* < .0001.

## Discussion

Experiment 2 confirmed Predictions 2.1 and 2.2: participants were more likely to confuse bare assertions with ‘seen’ claims in memory than vice versa without making similar confusions between ‘seen’ and ‘told’ claims. These results support the hypothesis that episodic assertions lacking an accompanying evidential claim tend to be interpreted as being based on first-hand evidence. This circumstance can explain why the participants’ believability judgments for bare assertions and claims to first-hand evidence looked similar in Experiments 1 and 2: they were sometimes interpreted to have the same evidential basis. One might object to this interpretation by pointing out that memory confusions could have occurred at retrieval rather than at encoding: That is, participants might have had a general bias to recall information as being based on claims to first-hand evidence (for a similar effect see Tosun, Vaid, & Geraci, 2013) without encoding bare assertions as ‘seen’ claims. Note that such a retrieval-based account would predict a general bias towards ‘seen’ claims independently of a statement’s original source. However, we only found a recall bias towards ‘seen’ claims for statements that had originally been barely asserted and not for ‘told’ claims.

More importantly, however, Experiment 2 also confirmed Prediction 2.3: When participants encoded a given statement as being associated with a claim to first-hand evidence, that statement was associated with higher believability than when they did not. We did not find a similar relationship between believability and ‘no source’ memory responses. It is not obvious how a purely retrieval-based interpretation of the memory confusion findings would explain this result. Instead, these results suggest that when the participants indeed interpreted (and encoded) a given statement as a claim to first-hand evidence, they also believed it more than when they did not encode such an evidential claim. Thus, when claims to first-hand evidence and bare assertions are encoded as such (i.e., when the latter is not confused with the former), they differ in their effect on statement believability.

Finally, Experiment 2 also confirmed that bare assertions increase the believability of a statement compared with its prior likelihood: bare assertions in Experiment 2 were associated with higher believability ratings compared with likelihood ratings for the same statements in Experiment 1.

## Experiment 3

Experiment 2 suggested that participants tended to interpret bare assertions as claims to first-hand evidence and that whether participants distinguished between these statement types in memory was predicted by how much they had initially believed it. Thus, Experiment 2 provided an explanation for why ‘seen’ statements and bare assertions behave similarly in terms of statement believability. However, the fact that bare assertions are implicitly interpreted as claims to first-hand evidence might also provide an explanation for why these claims behaved closely similar in terms of speaker accountability in Experiment 1. To test this hypothesis, Experiment 3 replicated the results of Experiment 2 and extended them to accountability judgments. Thus, we sought to test whether believability and accountability judgments would discriminate between whether participants responded with ‘seen’ or ‘no source’ in the memory test.

## Methods

### Participants

We recruited 200 native English-speaking participants from Testable Minds. As in the previous two experiments, we excluded 24 participants who provided the same response to all questions in one of the three tasks (believability judgments, memory judgments, or accountability judgments) as well as three participants who did not answer the majority of the math distractor questions correctly. Additionally, the data files of 14 participants were corrupted due to technical errors so that 159 participants were included in the final sample (*M*_Age_ = 35.90 years, *SD*_Age_ = 13.47 years, 75 females).

### Design

Experiment 3 was identical in design to Experiment 2, but also included the accountability judgment task from Experiment 1 after the memory test (see Fig. 1). That is, after the participants had made believability judgments about each statement, completed the math distractor task, and recalled the source claim associated with each statement, they were presented with each statement once again together with new information revealing that each speaker had been wrong. Crucially, unlike in Experiment 1, in the accountability judgement task, statements were not presented with their associated evidential claim (the complete procedure can be accessed here: https://www.testable.org/experiment/2878/185472/start).

Experiment 3, therefore, allowed us to test the following predictions:

- *Prediction 3.1:* Both believability and accountability judgments should discriminate between ‘seen’ and ‘bare assertions’ memory responses.
- *Prediction 3.2:* The association between believability and accountability judgments should be modulated by which source claim participants recalled a given statement to have been associated with.

## Results

### Replication of Experiment 2

Regarding participants’ responses in the memory task, the results of Experiment 3 were closely similar to those of Experiment 2. LRT-based model comparison between logistic mixed-effects models with random effects on the participant and vignette level (with fixed slopes) revealed that participants performed better at recalling ‘seen’ claims (*M*_Seen-Recall_ = 0.67, *SD*_Seen-Recall_ = 0.36) than ‘bare assertions’ (*M*_*A*sserted-Recall_ = 0.56, *SD*_Asserted-Recall_ = 0.33), χ^2^(1) = 12.12, *p* < .001, 95% CI [0.23, 0.82], while there was no difference in recall between bare assertions and ‘told’ claims (*M*_Told-Recall_ = 0.58, *SD*_Told-Recall_ = 0.39, *p* =.136).

Comparison of proportions of different memory error types (see Fig. 4b) to chance level (0.5) via one-sample *t* tests (with a Bonferroni-corrected alpha level of 0.017) showed that memory errors in the ‘told’ condition were roughly equally distributed between ‘seen’ (*M* = 0.52, *SD* = 0.47) and ‘no source’ (i.e., ‘bare assertion’) responses, *t*(95) = 0.32, *p* = 0.75. In contrast, participants were significantly more likely than chance to recall bare assertions as ‘seen’ (and less likely than chance as ‘told’) claims: similar to Experiment 2, an average proportion of 0.82 (*SD* = 0.3) of memory errors in bare assertion trials was due to participants confusing bare assertions with ‘seen’ claims, *t*(122) = 12.01, *p* < .0001. Participants were also more likely than chance to confuse ‘seen’ claims with bare assertions in memory (and less likely than chance to confuse them with ‘told’ claims; *M* = 0.71, *SD* = 0.44), *t*(81) = 4.2, *p* < .0001. However, a comparison to chance of the proportion of confusions between bare assertions and ‘seen’ claims that were due to bare assertions being recalled as ‘seen’ (*M* = 0.73, *SD* = 0.36) suggested that this was by far the more common memory error, *t*(135) = 7.46, *p* < .0001.

### Prediction 3.1: Did both believability and accountability judgments discriminate between ‘seen’ and ‘no source’ memory responses?

In order to assess whether believability and accountability judgments would predict ‘seen’ memory responses, we employed the same modeling approach as in Experiment 2. That is, we fit a binomial logistic mixed-effects model to a binary variable coding for whether participants had responded with ‘seen’ in the memory test, and another to a binary variable coding for whether they had responded with ‘no source’ in the memory test. Each model included statements’ original source (seen/told/asserted, dummy coded), participants’ believability judgments, and participants’ accountability judgments as fixed effects. As random effects, we included participant and statement number with fixed slopes.^7^^,^^8^

LRT-based model comparison suggested that (controlling for a statement’s original source) both believability, χ^2^(1) = 10.51, *p* = .001, 95% CI [0.06, 0.26], and accountability, χ^2^(1) = 3.93, *p* = .047, 95% CI [0.001, 0.19], ratings predicted participants’ ‘seen’ responses in the memory test. However, neither believability judgments, χ^2^(1) = 0.84, *p* = .358, 95% CI [−0.16, 0.06], nor accountability judgments, χ^2^(1) = 1.01, *p* = .315, 95% CI [−0.05, 0.15], predicted ‘no source’ responses.

### Prediction 3.2: Was the association between believability and accountability judgments modulated based on remembered source claims?

Finally, we computed separate linear regression models for the relationship between believability and accountability ratings based on what source claim participants remembered a given statement to have been associated with. The results of this analysis are summarized in Table 1 and suggest that Experiment 3 confirmed Prediction 3.2: believability and accountability ratings were associated whenever participants recalled a statement as ‘seen’ or ‘told’ but not when they recalled it to have been merely asserted. Thus, the association between believability and accountability depended to some extent on participants’ source claim recall.

**Table 1:** Predicting believability from accountability as a function of remembered and actual sources in Experiment 3

*Believability ~ Accountability*						
Trial type	Δ*R*^2^	β	*SE*	95% CI	*t*	*p*
Remembered Seen*	0.011	0.11	0.04	[0.02 – 0.19]	2.57	**.01**
Remembered Told**	0.021	0.159	0.06	[0.04 – 0.28]	2.63	**.009**
Remembered “No Source” (Bare Assertion)	-0.001	0.038	0.04	[-0.05 – 0.13]	0.85	.396

*Note.* The *p* values that survived Bonferroni-correction for multiple comparisons (.05/3 = .017) are printed in boldface.

## Discussion

Experiment 3 replicated the results of Experiment 2 and extended them to accountability judgments. While neither believability nor accountability ratings differed between originally presented ‘seen’ claims and bare assertions, both types of ratings predicted ‘seen’ memory responses without predicting ‘bare assertion’ memory responses. This suggests that when participants in fact distinguish between bare assertions and claims to first-hand evidence, they distinguish between them both in terms of how they impact statement believability and speaker accountability.

## General discussion

The current study set out to investigate whether source claims impact the believability of assertions purely through their function as arguments (i.e., by appealing to people’s intuitions about the reliability of different sources) or whether they also affect what listeners’ take a speaker to be committed to. We predicted that if source claims function as commitment devices, they should impact how strongly believability and accountability are associated with one another. More specifically, we asked two main research questions:

1. To what extent do claims to first-hand evidence increase, and claims to second-hand evidence decrease, statement believability?
2. To what extent are modulations in statement believability by source claims associated with analogous modulations in perceived speaker accountability?

In order to answer these questions, we compared the effects of claims to first-hand (“I saw that . . .”) and second-hand (“Somebody told me that . . .”) evidence to the effect of bare assertions about specific past events in terms of statement believability and speaker accountability.

### Source claims increase the association between believability and accountability

First, regarding Research Question 2, the main motivation for the present study was the investigation of the mechanism by which source claims modulate statement believability. We introduced a distinction between two possible routes by which evidential claims might have an impact on statement believability. On the one hand, listeners might have intuitions about the differential reliability of different sources grounded in a tacit theory about what happens to a piece of information as it is transmitted between cognitive systems. Listeners might assume that the more steps of inference a piece of information has undergone, the more likely it is that it has been corrupted. Thus, listeners might monitor the number of ‘inferential steps’ a piece of information has taken in order to arrive at them and adjust their belief in that information accordingly. On the other hand, while it seems plausible that listeners indeed might have such a tacit theory, it is unlikely that it would explain the entirety of the effect of evidential claims on statement believability. After all, talk is cheap and, as such, speakers might purport to base their claims on a certain evidential basis only to achieve a desired effect on believability.

Thus, the effect of a given source claim on believability must come at a potential cost to the speaker in the form of enhanced responsibility for the truth of their assertion (see, e.g., Vullioud et al., 2016). The effect of evidential claims on statement believability might therefore be driven by modulations in perceived speaker commitment, which in turn should express itself in how accountable a speaker is held for stating a falsehood. We investigated this idea by testing the prediction that ratings for statement believability and speaker accountability would be more strongly associated in the presence of an explicit evidential claim. Both Experiments 1 and 3 confirmed this prediction: Explicit evidential claims increased the association between believability and accountability ratings. While this result is compatible with the idea that source claims modulate statement believability partially through modulating speaker accountability, the correlational nature of the current study does not allow us to draw strong conclusions on this point. It is, for example, also possible that modulations in statement believability drove how accountable a speaker was perceived to be for its truth. Future studies should therefore seek to establish a causal relationship between speaker accountability and statement believability not mediated by source claims. Nonetheless, the results presented here tie in with the idea that tracking where one’s own beliefs come from is not just important for epistemic reasons (e.g., Mahr & Csibra, 2018; 2020; Mahr et al., 2020). Instead, source information plays an important role when one wants to transmit those beliefs to others by allowing speakers to calibrate their conversational commitments.

### The effect of claims to first- and second-hand evidence on statement believability

Regarding the question of the effect of source claims on statement believability (Research Question 1, above), we found that claims to second-hand evidence consistently decreased both statement believability as well as speaker accountability compared with claims to first-hand evidence and bare assertions (Experiments 1–3). This result might be construed to contradict findings by Collins and Hahn (2019) who found that indirect evidentials (“I suspect that . . .”) did not protect a speaker’s reputation from damage. However, Collins and Hahn did not use explicit claims to second-hand evidence. Indirect evidentials might thus be less effective at “hedging one’s bets” and affect speaker commitment less than the less committal reportative claims we used in the present study (“Somebody told me . . .”).

The effect of claims to first-hand evidence both on believability and accountability was less clear-cut: In Experiment 1, claims to first-hand evidence and bare assertions behaved closely similar both in terms of their effects on statement believability and speaker accountability. On the one hand, such a similarity might be expected given that claims to first-hand evidence and bare assertions should commit the speaker to the embedded proposition and therefore influence a statement’s believability and speaker accountability in the same direction. Indeed, we found evidence that merely being asserted increased a statement’s believability compared with how likely people would have thought it to be true otherwise (Experiments 1 & 2).

On the other hand, however, Experiments 2 and 3 showed that participants tended to recall bare assertions as claims to first-hand evidence. This suggests that they interpreted bare assertions as not only committing the speaker to the embedded proposition but also to having good evidence about it. This finding thus adds to an existing literature that memory encoding privileges implied meanings over explicit utterances (Brewer, 1977; Chan & McDermott, 2006). Moreover, it suggests that bare assertions about episodic claims are routinely interpreted as claims to first-hand evidence; a result in line with findings by Degen et al. (2019). This effect, which we did not expect to find when we launched this study, likely confounded our comparison between claims to first-hand evidence and bare assertions.

Nonetheless, this finding ties in with several observations in the literature on evidentials. On the one hand, it has been found that listeners commonly infer from a speaker’s use of a ‘more indirect’ evidential that she was not in a position to provide a ‘more direct’ one (Faller, 2001; Papafragou et al., 2007). Consequently, speakers might not have to make explicit when they have direct evidence but only when they do not have such evidence. On the other hand, languages with evidential systems generally tend to mark indirect compared with direct information access: while the World Atlas of Languages (de Haan, 2013) lists 166 languages that exclusively mark indirect evidentials, and 71 languages that mark both direct and indirect evidentials, it does not list any languages only marking direct evidentials. The finding that bare assertions are commonly interpreted as claims to direct perceptual evidence is also in line with results from learnability experiments for evidentials (Saratsli et al., 2020). Saratsli et al. (2020) found that reportative evidentials are easier to learn than evidentials marking direct perceptual access. One explanation these authors offered for this result was that perception might be viewed as a ‘default’ mode of evidence and therefore would be less informative to encode explicitly. The fact that participants in our experiments tended to treat bare assertions as claims to first-hand evidence supports this idea.

However, Experiments 2 and 3 accounted for this confound between bare assertions and ‘seen’ claims. The results from these experiments suggested that the extent to which such a source claim is made explicit makes a difference in terms of how believable a statement and how accountable for its truth its speaker is taken to be (Bonalumi et al., 2020): Our participants’ believability and accountability judgments differentiated between statements they remembered to have been asserted on the basis of first-hand evidence from those remembered to have been asserted on other grounds.

All in all, these results support the notion that whenever participants took a speaker to make an assertion on the basis of first-hand evidence, they took it to be more believable, while claims to second-hand evidence had the opposite effect. Note, though, that we tested these claims on a narrowly circumscribed set of materials, and therefore it remains to be determined to what extent these findings generalize to more naturalistic contexts. Nonetheless, these results provide direct experimental evidence for the intuitive notion that claiming first-hand evidence causes people to treat a statement as more believable while claiming second-hand evidence causes them to treat it as less believable.

### A ‘Kuzari effect’?

The medieval Jewish philosopher Judah Halevi famously argued in the ‘Kuzari’ that the oral testimony of the story of the Jewish Exodus from Egypt itself proves that it must have occurred. In effect, according to Halevi, the fact that the entirety of the Jewish people believed that the Exodus from Egypt had occurred, should be reason for us to believe so, too.

Reminiscent of this idea, in Experiments 1 and 2 we found that participants believed a claim more after it had been asserted compared with how likely they had judged it to be beforehand. On the one hand, this effect might be due to the fact that asserting a claim provides evidence to listeners that at least one other person believes that claim. This might contribute to making it more believable. On the other hand, an assertion might make a claim more believable because making an assertion should commit the speaker to its truth, and listeners might take the fact that a speaker takes responsibility for the truth of a claim as a reason to believe it. While we could not disentangle these two explanations in the current study, future work should seek to do so.

## Footnotes

1 We thank an anonymous reviewer for making some clarifying suggestions on this point.

2 Of course, it is worth mentioning that evidential claims likely *do* have epistemic functions that are independent of their effect on speaker commitment. For example, source tags might provide background information about what else the speaker might know (e.g., Faller, 2002; Nagel, 2015). Moreover, while evidential claims are akin to expressions of certainty, they differ in their flexibility: While one can freely modulate one’s expressed degree of certainty, changing what source one claims to have might be viewed as lying. Thus, we are not claiming here that evidential claims are identical to modal expressions. Rather, we propose that modal and evidential expressions sometimes achieve epistemic effects in similar ways—namely, through the modulation of perceived speaker commitment.

3 Note that it would be possible for accountability assessments to drive believability judgments in spite of the fact that, in the current study, we measure accountability after believability. This delayed measurement is due to the circumstance that our method for measuring accountability requires participants to receive new information that reveals the speaker’s statement to be false. Nonetheless, participants might compute a speaker’s commitment when they decide how much to believe their assertion (that is, when we measure believability) which in turn should drive their accountability judgments.

4 In contrast to believability and likelihood judgments, accountability judgments showed both a higher number of extreme (i.e., maximal) responses as well as a higher number of zeroes in difference scores. Because this suggested that participants had used the rating scales differently in making likelihood, believability, and accountability judgments, respectively, we computed marginal entropies (with the use of the *entropy* R package, Hausser & Strimmer, 2009) for likelihood (*H*_Likelihod_ = 2.21), believability (*H*_Believability_ = 2.24), and accountability difference scores (*H*_Accountability_ = 2.01) to test whether they contained a comparable amount of variability. Marginal entropy can be treated as a measure of the amount of variability contained within a given dataset. The fact that marginal entropies for our measures were similar suggests that all measures contained a comparable amount of variability.

5 Because each participant contributed only one difference score per source contrast and vignette, mixed models did not converge for these analyses. For this reason, we chose the generalized linear model approach instead.

6 This effect could be due to the fact that making an assertion provides evidence to a listener that at least one person believes the claim in question or that participants had already been exposed to each statement before making a believability assessment about these assertions. Experiment 2 could rule out the latter interpretation (i.e., that the increased believability of bare assertions might have been a mere ‘repeated-exposure’ effect).

7 We used statement rather than vignette number here because participants were presented with individual statements rather than the entire vignette setting in the accountability judgment task.

8 Because a model with a two-tiered random effect structure did not converge for ‘no source’ memory responses, we dropped the item (i.e., statement) level random effect for this model.
