# Correction to: The effect of source claims on statement believability and speaker accountability

**DOI:** 10.3758/s13421-021-01215-9

**Published:** 2021-07-29

**Authors:** Johannes B. Mahr, Gergely Csibra

**Affiliations:** 1 Department of Psychology, Harvard University, 33 Kirkland St, Cambridge, MA 02138 USA; 2 Department of Cognitive Science, Central European University, Budapest, Hungary; 3 Department of Psychological Sciences, Birkbeck, University of London, London, UK

**Correction to: Memory & Cognition.**

10.3758/s13421-021-01186-x

The article “The effect of source claims on statement believability and speaker accountability”, written by Johannes B. Mahr and Gergely Csibra, was originally published electronically on the publisher’s internet portal on 14 June 2021 without open access. With the author(s)’ decision to opt for Open Choice, the copyright of the article changed on 23 June 2021 to © The Author(s) 2021 and the article is forthwith distributed under a Creative Commons Attribution 4.0 International License, which permits use, sharing, adaptation, distribution and reproduction in any medium or format, as long as you give appropriate credit to the original author(s) and the source, provide a link to the Creative Commons license, and indicate if changes were made. The images or other third party material in this article are included in the article’s Creative Commons license, unless indicated otherwise in a credit line to the material. If material is not included in the article’s Creative Commons license and your intended use is not permitted by statutory regulation or exceeds the permitted use, you will need to obtain permission directly from the copyright holder. To view a copy of this license, visit http://creativecommons.org/licenses/by/4.0/.

